# 3D printer audio and vibration side channel dataset for vulnerability research in additive manufacturing security

**DOI:** 10.1016/j.dib.2024.111002

**Published:** 2024-10-10

**Authors:** Christos Madamopoulos, Nektarios Georgios Tsoutsos

**Affiliations:** a Department of Electrical and Computer Engineering, University of Delaware, Newark, DE, USA; b Department of Electrical and Computer Engineering, National Technical University of Athens, Zografou, Greece

**Keywords:** Side channels, Side channel attacks, Additive manufacturing, 3D printing, Cyber physical systems, Cybersecurity

## Abstract

This dataset provides a comprehensive set of side channels from fused deposition modeling 3D printers in order to enable the research in the security of additive manufacturing processes against side channel attacks. These attacks exploit indirect signal emanations from physical processes to extract information about a system. Our data was collected using two different methods (iPhone app and Teensy 4.0 sensor system) on two different 3D printers (Bambu Lab P1P and A1 mini), and consists of two types of data, audio data in the form of the recording of the 3D printer's sound while printing, and vibration data in the form of the linear acceleration in the cartesian coordinates. The dataset includes data from 12 different 3D objects that cover a wide variety of movements made while 3D printing. Along with the side channels this dataset includes the source computer-aided design files of the objects, as well as .gcode and .3mf files used by the printers.

Specifications Table

**Table:** 

Subject	Computer Science, Signal Processing.
Specific subject area	Additive Manufacturing; Cyber physical systems; Side channels; Signal Processing.
Type of data	.mp3 (audio file), .caf (audio file), .mp4 (video file), .csv (accelerometer readings), .3mf (3D model sliced file), .gcode (3D model sliced file), .stl (3D model file), .pdf (table of contents).
Data collection	A total of 144 audio and 144 vibration recordings were recorded over a 4-week period by two different systems. The first one uses a teensy 4.0 microcontroller and the other one uses an iPhone. Two different 3D printers (make: Bambu Labs models: P1P and A1 mini) were used to fabricate twelve different 3D artifacts. The artifacts were selected to cover a diverse range of shapes and motion of the 3D printers. The audio files are the sound of the printer while in operation, and the vibration files give the linear acceleration of the x, y, z axes caused by the printer vibrations.
Data source location	University of Delaware, Department of Electrical and Computer Engineering, Newark, DE, USA.
Data accessibility	The dataset is available for anonymous access at zenodo.org Repository name: 3D printer audio and vibration side channels Digital Object Identifier (DOI): 10.5281/zenodo.13329934 Direct URL to data: https://zenodo.org/records/13329934 The dataset is available for browsing and download at the link above.
Related research article	None.

## 1 Value of the Data

• The data provides the means by which the scientific community will be able to explore the vulnerabilities of additive manufacturing to side channel attacks, based on audio and vibration side channels.
• This data covers a wide variety of 3D models, so that all the usual movements of a printer head are well documented. Due to the broadness and scale of the dataset, it will be able to provide insight on if certain objects are at greater risk than others to malicious actors implementing side channel attacks.
• The side channel data is collected from two very recent and widely used 3D printers, which have side channel protection (i.e., motor noise cancellation, anti-vibration technology). These printers are of the core-XY, or the cartesian (a.k.a. bedslinger) variety, which are the most used 3D printer varieties.
• The side channels are provided along with the 3D designs and sliced files in order to enable the reverse engineering of the 3D objects from their respective side channels.

## 2 Background

Securing the cyberspace has evolved into one of the most complex engineering challenges we face today. With the increasing integration of cyber-physical systems into various industries, the threat landscape has expanded significantly. Among these threats, Side Channel Attacks (SCAs) have emerged as a particularly insidious risk. SCAs exploit indirect signal emanations from physical processes to extract sensitive information about a system, posing a substantial threat to the integrity and security of cyber-physical systems. One prominent example of a cyber-physical process vulnerable to such attacks is additive manufacturing, particularly 3D printing. As the 3D printing industry grows at a rapid pace, it is transitioning from being confined to individual manufacturing facilities or a small set of trusted parties (operating within a secure environment) to becoming a digital manufacturing ecosystem. In this ecosystem, different entities handle various steps of the process, leading to increased cybersecurity risks [1]. The distributed nature of modern additive manufacturing systems makes them more susceptible to SCAs, as the attack surface expands with each additional entity involved.

While it has been demonstrated that 3D parts can be authenticated through non-fungible tokens (NFTs) [2], this method does not entirely eliminate the risk of counterfeiting through SCAs. Our dataset aims to address this gap by providing a comprehensive set of side channels from fused deposition modeling 3D printers. By focusing on acoustic and vibration side channels, which attackers can easily acquire with low risk of detection, our data helps identify characteristics that enable potential theft and counterfeiting. Previous research has primarily focused on a limited variety of 3D models [3], restricting the scope of analysis and potential countermeasures. In contrast, our dataset is the first to provide acoustic and vibration data for a larger sample of models with diverse geometrical features (e.g., straight, circular, arced toolpaths). This diversity allows for a more thorough examination of the vulnerabilities in 3D printing processes. Additionally, our dataset includes the source computer-aided design (CAD) files of the objects, as well as .gcode and .3mf files used by the printers. This comprehensive approach enables researchers to reverse engineer the original 3D design files, furthering our understanding of how SCAs can be mitigated in the context of additive manufacturing. By providing this dataset, we aim to contribute to the ongoing efforts to secure digital manufacturing processes against SCAs. As the industry continues to evolve, it is crucial to develop robust security measures that can protect against these sophisticated attacks, ensuring the integrity and reliability of 3D printed parts in various applications.

## 3 Data Description

The dataset consists of 288 different data files. These are based on two different 3D printers and two different data acquisitions devices. In particular, the Bambu Labs P1P and Bambu Labs A1 mini 3D printers are used for the fabrication of different 3D artifacts, whereas an iPhone and a teensy microcontroller system are used to collect the data. The term *artifact* is used to describe the 3D design of each model that has been printed. Each artifact was printed three times. In this data in brief, the .stl and .gcode/.3mf files used to print the artifacts are provided in the dataset. Further description for each of the artifacts and the side channels is below.

### 3.1 Artifact description

For our dataset, we employed 12 different 3D artifacts, which are detailed in the following paragraphs. Our motivation for selecting these models was threefold: (i) to encompass a wide variety of motions characteristic of 3D printers (e.g., straight lines, curves, and combinations thereof), (ii) to include standardized artifacts, and (iii) to incorporate artifacts that can clearly demonstrate the potential consequences when side channel information is leaked.

**Fig. 1:**
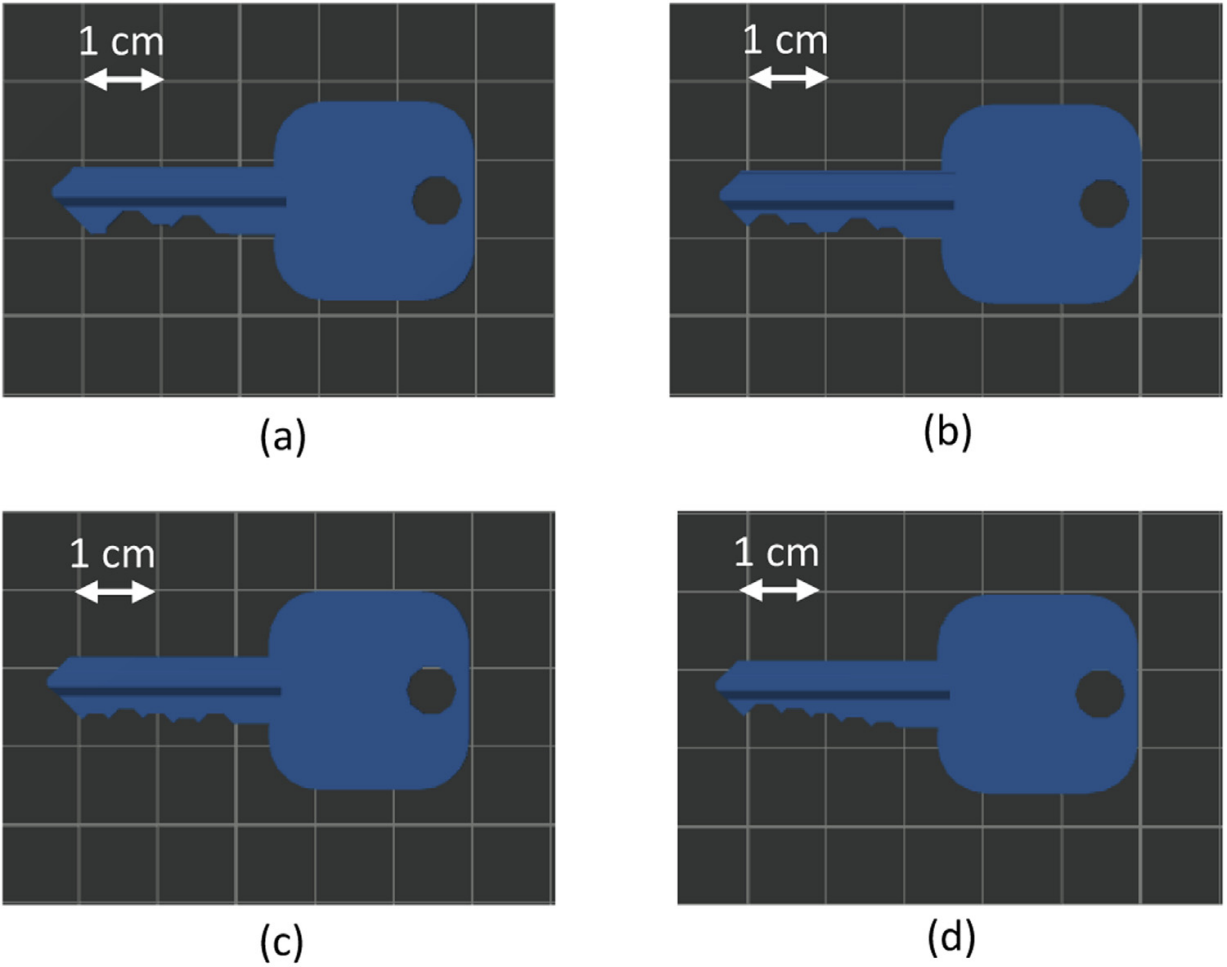
(a) e*asy key*, (b) m*edium key*, (c) h*ard key*, (d) s*teps key*.
(b) Artifact 2, *Medium key* (Fig. 1b): Adjacent notch depths have either large (i.e., ±0.046”) or small (i.e., ±0.023”) differences according to [6]. This object aims to help differentiate between the large and small cuts.
(c) Artifact 3, *Hard key* (Fig. 1c): Adjacent notch depths have a small difference (i.e., ±0.023”) according to [6]. This object aims to help differentiate between the increase or decrease of each cut, at the small resolution of 0.023”.
(d) Artifact 4, *Steps key* (Fig. 1d): Adjacent notch depths have a difference of −0.023” according to [6]. This object aims to provide all acceptable depths in a descending order (from −0.023” to −0.115”)

The first four artifacts represent keys based on the OpenSCAD design of a Kwikset KW1 5 pin key [4,5] and are categorized as *easy, medium, hard* and *steps*. In particular, the feature size and direction, and the goal of each design is:

(a) Artifact 1, *Easy key* (Fig. 1a): Adjacent notch depths have a large difference (i.e., ±0.046”) according to [6]. This object aims to help differentiate between the increase or decrease of each cut, at a resolution of 0.046”.

The goal of the key artifacts is to be able to test the resolution of the side channels. It is important to note that the differences in the key characteristics are very subtle and pose a significant security risk if it is possible to recreate the exact layout of the keys.

**Fig. 2:**
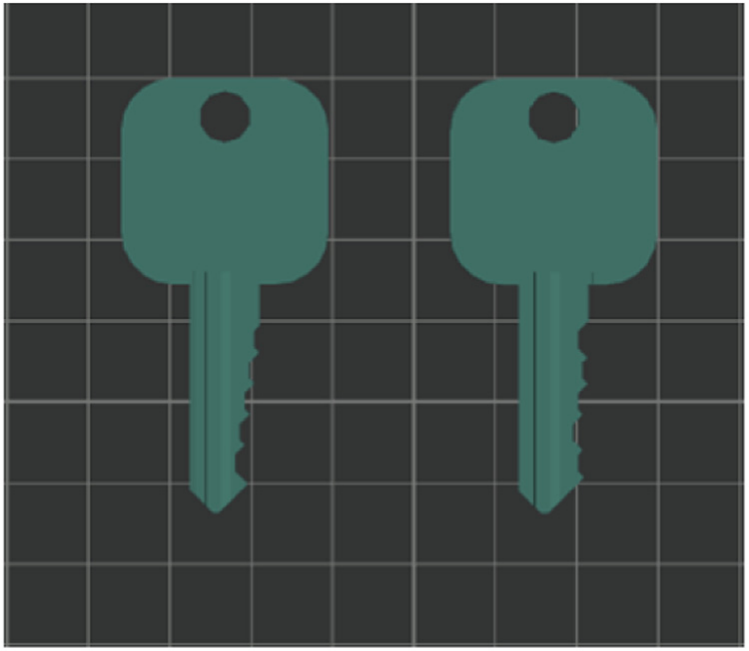
Two keys artifact, hard key (right) steps key (left).

The 5th artifact is a single print of the *hard key* and the *steps key*. The difference compared to the previous keys is in the printing implementation. In particular, the printer prints both key layers before moving on to the next layer. Each layer begins with the *hard key* and ends with the *steps key.* The goal is to test if it is possible to distinguish between the two singular layers using the side channels. The artifact is named *two keys*. The slicing settings for all the key designs are the 0.16 mm High Quality Bambu Labs preset (Fig. 2).

**Fig. 3:**
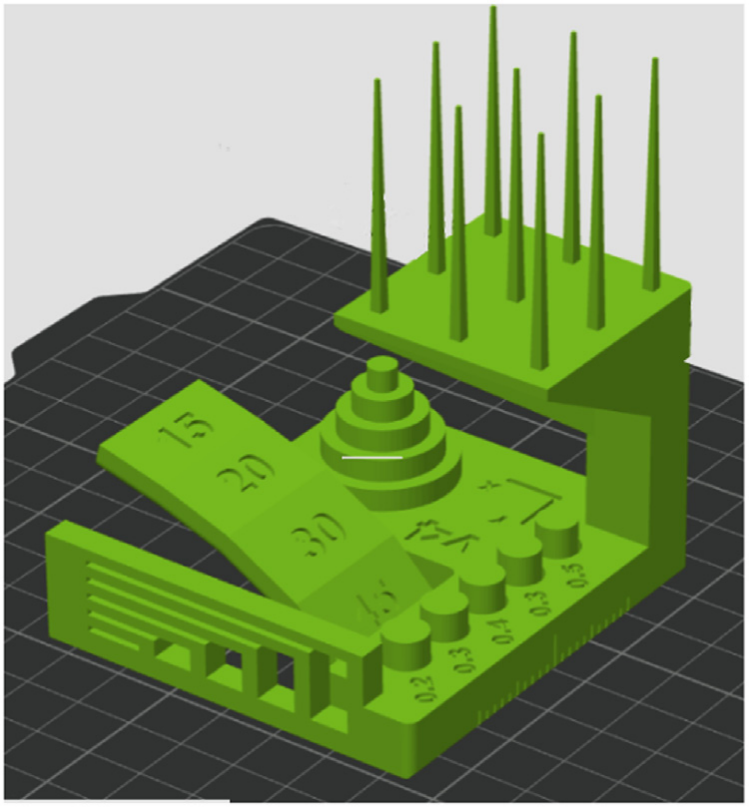
The Autodesk and Kickstarter FDM 3D printer assessment model.

The 6th artifact is the *Autodesk and Kickstarter FDM 3D printer assessment model*, which is used to benchmark 3D printer projects on Kickstarter. The model tests dimensional accuracy, negative feature resolution, positive feature resolution/fine flow control, basic overhang capabilities, basic bridging capabilities, XY ringing, Z-axis alignment [7]. The slicing setting was the 0.16 mm High Quality Bambu Labs preset (Fig. 3).

**Fig. 4:**
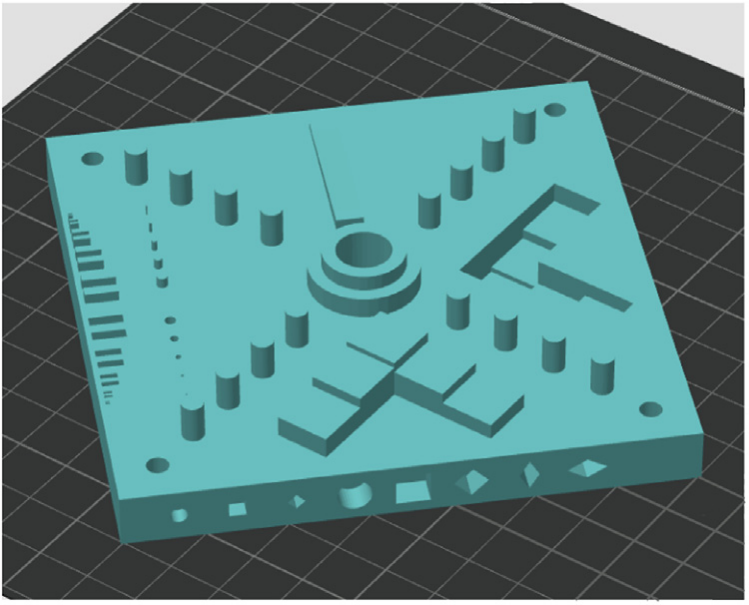
National Institute of Standards and Technology (NIST) additive manufacturing test artifact.

The 7th model is the *National Institute of Standards and Technology (NIST) additive manufacturing test artifact* [8], that investigates straight features, parallel or perpendicular features, circular or arced features, concentric circles or arcs, fine features, 3D or freeform features, holes and bosses, multiple planes, location and orientation, geometric errors of mirror positioning, geometric errors of build platform, alignment errors between axes, and errors in beam size. The slicing setting for this artifact was the 0.16 mm High Quality Bambu Labs preset (Fig. 4).

**Fig. 5:**
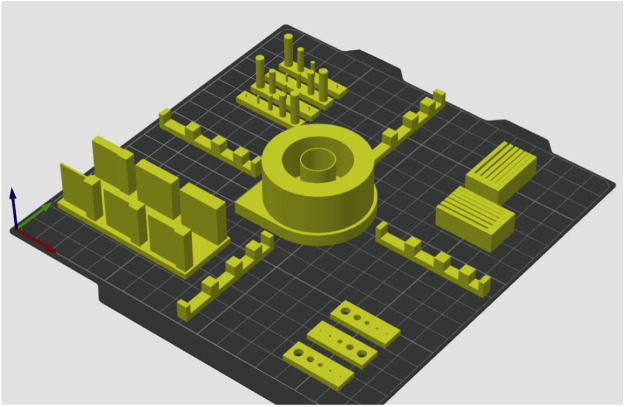
The ASTM/ISO 52902 artifact model.

The 8th model adopts the specifications outlined in the *ASTM/ISO 52902 standards* [9]. Specifically, in the model there are four linear artifacts, one circular artifact, four resolution pin artifacts, two resolution slot artifacts, three resolution hole artifacts, and one resolution rib artifact. The slicing setting for this artifact was the 0.16 mm High Quality Bambu Labs preset (Fig. 5).

**Fig. 6:**
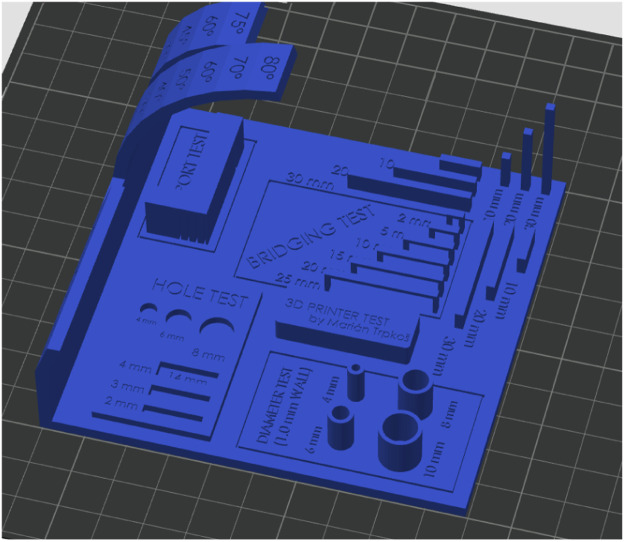
All in one test artifact.

The 9th artifact is the *all in one test* [10], it is one of the most popular tests to evaluate the capabilities of 3D printers. This test includes a support test, scale test, overhang test, hole test, diameter test and bridging test. The slicing setting for this artifact was the 0.16 mm High Quality Bambu Labs preset (Fig. 6).

**Fig. 7:**
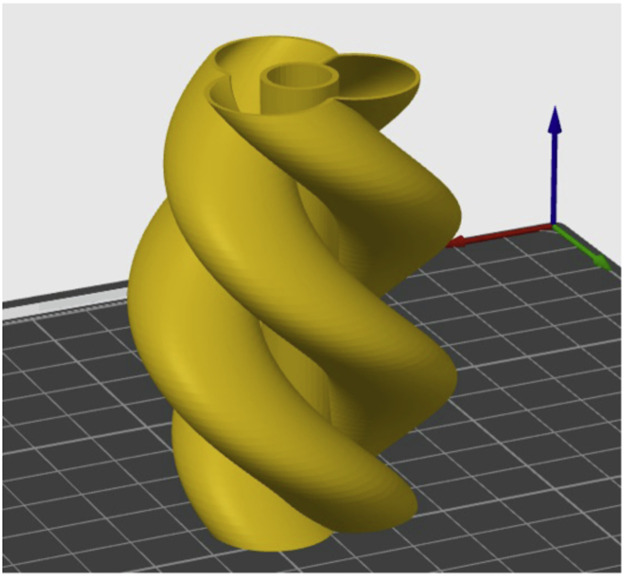
Triple helix artifact.

The 10th artifact is a *triple helix* vase [11] design. This object was chosen in order to replicate some bioprinting applications with its swooping hollow curves and overhang elements. The slicing setting for this artifact was the 0.16 mm High Quality Bambu Labs preset, at 70% scaling (Fig. 7).

**Fig. 8:**
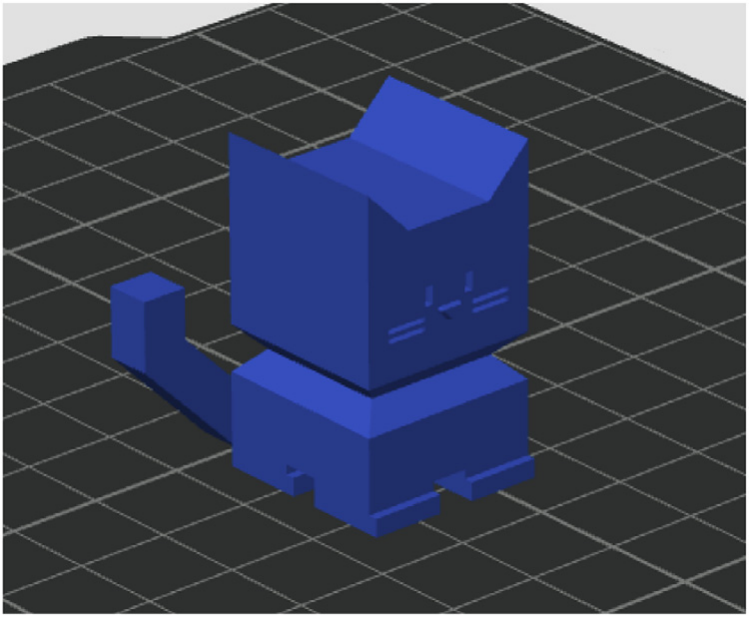
Calibration cat artifact.

The 11th artifact is called a *calibration cat* (*CaliCat*) [12], this object is made up of rectangular and triangular components, and our motivation is to have a recording that captures exclusively straight lines. The slicing setting for this artifact was the 0.16 mm High Quality Bambu Labs preset, with the infill type set to rectilinear (Fig. 8).

**Fig. 9:**
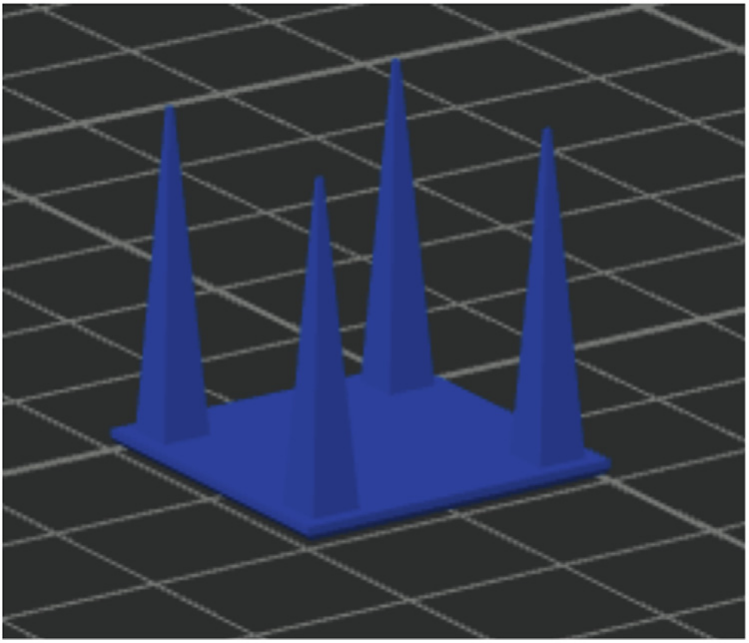
Retraction test artifact.

The 12th and final artifact is called a *retraction test* [13]. This object is made up of four vertical spikes on a square base, the goal of this object is to capture the side channels that correspond to the retraction of the print head from an object. The slicing setting for this artifact was the 0.16 mm High Quality Bambu Labs preset (Fig. 9).

### 3.2 Dataset statistics

In this section, we provide some general information about the dataset with regard to the characteristics of the different elements of this dataset. Specifically, the characteristics of the .stl files are presented in Table 1. The characteristics of the sliced file and the printing of the object are presented in Table 2. In the remaining tables the specific characteristics of the audio data and vibration data are presented for each set of data (Table 3, Table 4, Table 5, Table 6, Table 7, Table 8).

**Table 1:** Geometric features of the artifact .stl files.

File Name	Number Of Triangles	Average Triangle Area (cm^2^)	Footprint (cm^2^)	Height (cm)	File Size (KB)
1_key_easy.stl	324	0.0617	13.6088	0.196	15.9023
2_key_medium.stl	320	0.0621	13.6088	0.196	15.7070
3_key_hard.stl	320	0.0629	13.6088	0.196	15.7070
4_key_steps.stl	320	0.0621	13.6088	0.196	15.7070
5_2keys_.stl	640	0.0625	33.0054	0.196	31.3320
6_ksr_fdmtest_v4.stl	12234	0.0244	52.5000	9.200	597.4453
7_NIST Test Artifact online.STL	7392	0.0391	200.0001	1.700	361.0195
8_parts_of_ASTM52902.stl	13860	0.0318	306.2500	2.600	676.8398
9_3D_Printer_test_fixed_stl_3rd_gen.STL	63622	0.0067	100.0000	6.052	3106.6250
10_Triple_Helix.stl	113036	0.0064	57.2554	10.460	5519.4180
11_calicat.stl	876	0.0518	8.1326	3.500	42.8555
12_four_square_cons.stl	82	0.1636	4.0000	2.000	11.0537

**Table 2:** Characteristics of the sliced file for each artifact (LoC: lines of code).

Artifact No	A1mini					P1P				
	GCode LoC	File (KB)	Number of layers	Filament spent (g)	Print time	GCode LoC	File (KB)	Number of layers	Filament spent (g)	Print time
1	9741	67	12	1.67	5m 33s	9158	227	12	1.64	4m 57s
2	9712	66	12	1.61	5m 32s	9100	225	12	1.63	4m 57s
3	9741	68	12	1.63	5m 31s	9068	226	12	1.65	4m 55s
4	9766	68	12	1.62	5m 32s	9114	227	12	1.63	4m 56s
5	17969	116	12	3.18	10m 29s	17139	409	12	3.18	9m 34s
6	386648	1856	575	36.65	2h 43m	363162	8888	575	36.65	2h 26m
7	250681	1449	106	47.59	2h 21m	247216	6990	106	47.52	2h 9m
8	338187	1771	162	51.57	3h 10m	345818	7902	162	54.4	3h 12m
9	331091	1568	378	48.46	2h 50m	314634	7028	378	48.47	2h 48m
10	484609	3301	457	15.78	1h 35m	462120	11300	457	15.8	1h 28m
11	45167	124	216	6.73	31m 34s	36380	833	216	6.74	33m 4s
12	23185	83	123	0.86	9m 55s	18305	409	123	0.88	7m 1s

**Table 3:** Audio and vibration file characteristics, on Bambu Labs P1P, for the **first replica** of each artifact.

Artifact No	P1P							
	iPhone				Teensy 4.0			
	Audio file size (MB)	Audio duration (min)	Vibration .csv rows	Vibration file size (MB)	Audio file size (MB)	Audio duration (min)	Vibration .csv rows	Vibration file size (MB)
1	2.39	5.23	32869	2.33	4.78	5.23	149717	3.26
2	2.49	5.46	34301	2.43	4.98	5.45	163560	3.58
3	2.43	5.3	33181	2.35	4.84	5.3	158814	3.47
4	2.48	5.41	34679	2.45	4.94	5.41	162321	3.5
5	4.66	10.2	64410	4.56	9.23	10.1	303234	6.61
6	68.1	148	891926	63.1	135	147	4422386	102
7	56.4	124	742279	52.5	113	124	2424999	55
8	83.2	183	1045509	73.9	167	183	5486472	127
9	77.7	170	994299	70.3	155	170	5090485	117
10	37.8	83.4	487082	34.4	76.1	83.3	2498883	57.7
11	16	34	207574	14.7	30.9	33.8	1015013	22.9
12	3.63	7.69	50541	3.57	6.97	7.63	228909	4.99

**Table 4:** Audio and vibration file characteristics, on Bambu Labs A1 mini, for the **first replica** of each artifact.

Artifact No	A1 mini							
	iPhone				Teensy 4.0			
	Audio file size (MB)	Audio duration (min)	Vibration .csv rows	Vibration file size (MB)	Audio file size (MB)	Audio duration (min)	Vibration .csv rows	Vibration file size (MB)
1	3	6.5	27788	1.97	5.96	6.52	195546	4.39
2	2.88	6.24	26337	1.87	5.99	6.55	196513	4.25
3	2.92	6.33	26043	1.85	5.63	6.16	184850	4.14
4	2.91	6.3	24273	1.36	5.85	6.4	190809	4.31
5	5.24	11.4	46605	3.31	10.3	11.2	334906	7.57
6	747	148	920878	66.2	135	148	4439033	104
7	60.7	133	841933	59.8	121	133	3986708	93.4
8	86.4	188	1155693	82	171	187	5606511	132
9	76.1	166	1019271	72.4	150	165	4936086	116
10	39.3	86.6	519003	36.8	79.1	86.6	1575001	37
11	13	27.9	106386	7.55	25.3	27.7	827581	19
12	3.85	8.31	35267	2.5	7.76	8.49	254514	5.75

**Table 5:** Audio and vibration file characteristics, on Bambu Labs P1P, for the **second replica** of each artifact.

Artifact No	P1P							
	iPhone				Teensy 4.0			
	Audio file size (MB)	Audio duration (min)	Vibration .csv rows	Vibration file size (MB)	Audio file size (MB)	Audio duration (min)	Vibration .csv rows	Vibration file size (MB)
1	2.45	5.35	33660	2.38	4.86	5.32	190247	4.26
2	2.44	5.33	34898	2.47	4.86	5.32	188504	4.07
3	2.41	5.26	33020	2.33	4.78	5.24	193767	4.38
4	2.49	5.34	34612	2.45	4.86	5.32	190868	4.3
5	4.7	10.22	64065	4.53	9.26	10.13	416384	9.38
6	67.78	147.61	862568	60.95	134.81	147.59	4448199	103.74
7	56.44	123.89	751193	53.11	113.16	123.89	3973675	92.97
8	83.2	182.89	1072606	75.81	167.04	182.87	5608080	132.35
9	77.76	169.83	1049178	74.14	155.07	169.77	4933153	115.95
10	37.76	83.24	486530	34.41	76.05	83.26	2611069	61.67
11	16.01	34.03	203975	14.44	31.06	34	805923	18.61
12	3.59	7.62	47798	3.38	6.93	7.58	228224	5.09

**Table 6:** Audio and vibration file characteristics, on Bambu Labs A1 mini, for the **second replica** of each artifact.

Artifact No	A1 mini							
	iPhone				Teensy 4.0			
	Audio file size (MB)	Audio duration (min)	Vibration .csv rows	Vibration file size (MB)	Audio file size (MB)	Audio duration (min)	Vibration .csv rows	Vibration file size (MB)
1	2.72	5.9	23277	1.65	5.82	6.37	151839	3.31
2	3.01	6.54	25678	1.82	5.74	6.29	159592	3.49
3	2.81	6.09	23732	1.69	5.9	6.46	156988	3.44
4	2.93	6.34	24449	1.74	5.81	6.36	159493	3.44
5	5.18	11.26	48183	3.42	12.68	13.88	304021	6.64
6	67.81	148.49	927660	65.86	135.5	148.34	4427166	101.1
7	60.77	132.79	826577	58.6	121.01	132.48	3715714	85.3
8	86.55	187.87	1108358	78.6	170.81	187	5484764	126.17
9	75.47	164.29	976096	69.25	150.24	164.48	5091676	116.86
10	39.47	87.07	527624	37.41	79.52	87.05	2497401	57.49
11	12.76	27.75	115740	8.22	24.92	27.28	1019897	22.99
12	3.9	8.39	33685	2.39	6.97	7.63	227431	4.96

**Table 7:** Audio and vibration file characteristics, on Bambu Labs P1P, for the **third replica** of each artifact.

Artifact No	P1P							
	iPhone				Teensy 4.0			
	Audio file size (MB)	Audio duration (min)	Vibration .csv rows	Vibration file size (MB)	Audio file size (MB)	Audio duration (min)	Vibration .csv rows	Vibration file size (MB)
1	2.5	5.44	34194	2.42	4.99	5.46	163900	3.56
2	2.56	5.53	34609	2.45	4.99	5.46	163900	3.56
3	2.52	5.48	34437	2.44	5.03	5.51	165305	3.59
4	2.51	5.49	34606	2.45	5.03	5.5	165061	3.59
5	4.62	10.13	63413	4.49	9.25	10.12	303647	6.64
6	67.15	147.61	898328	63.52	155.13	169.83	5093324	117.14
7	56.25	123.79	716352	50.65	113.09	123.8	3713440	84.9
8	83.06	182.89	1093252	77.26	167.02	182.85	5484015	125.99
9	77.16	169.82	984960	69.61	155.13	169.83	5093324	117.14
10	37.91	83.21	488138	34.53	76.03	83.23	2496847	57.1
11	15.44	33.95	206322	14.59	31.03	33.97	1019045	22.89
12	3.59	7.73	48519	3.43	7.08	7.75	232604	5.08

**Table 8:** Audio and vibration file characteristics, on Bambu Labs A1 mini, for the **third replica** of each artifact.

Artifact No	A1 mini							
	iPhone				Teensy 4.0			
	Audio file size (MB)	Audio duration (min)	Vibration .csv rows	Vibration file size (MB)	Audio file size (MB)	Audio duration (min)	Vibration .csv rows	Vibration file size (MB)
1	4.13	9.06	56660	4.02	8.27	9.05	271574	6.17
2	3.93	8.61	57454	4.07	7.86	8.61	258212	5.91
3	4.08	8.96	56394	4	8.1	8.86	265775	6.09
4	4.03	8.83	55506	3.94	8.08	8.84	265309	6.06
5	6.28	13.81	86479	6.13	12.62	13.82	414538	9.4
6	68.87	151.07	934129	66.17	137.97	151.04	4530128	107.05
7	60.3	133.04	818417	58	121.52	133.04	3990017	94.23
8	85.13	187.89	1152472	81.66	171.6	187.86	5632725	134.14
9	74.5	164.4	1025850	72.73	150.14	164.37	4928180	116.79
10	39.72	86.9	549217	38.93	79.37	86.89	2606419	61.49
11	13.86	30.53	186525	13.23	27.88	30.52	915567	21.33
12	5.09	11.17	73535	5.21	10.2	11.17	334893	7.66

### 3.3 Final data format and file structure

**Fig. 10:**
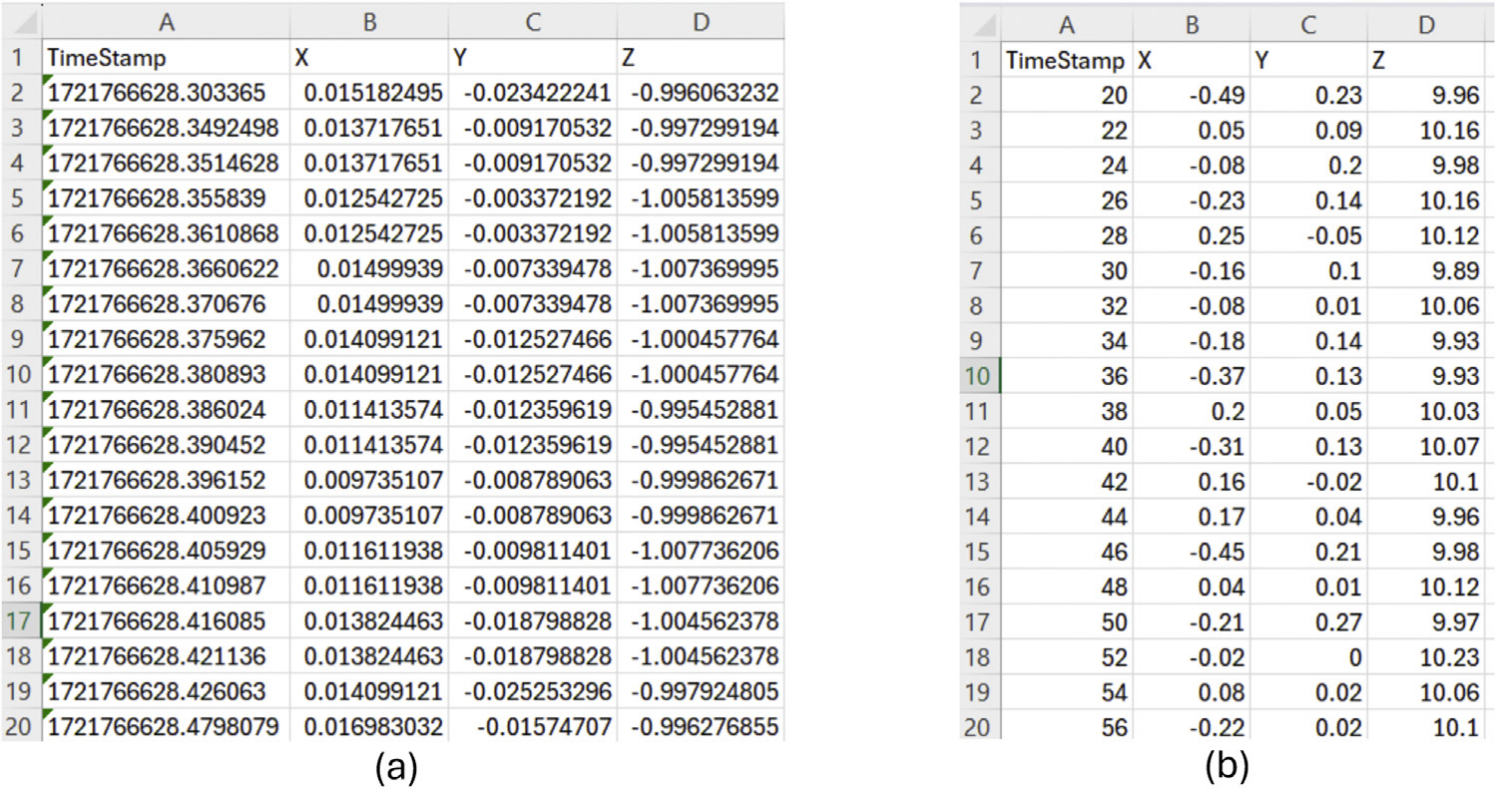
Screenshots of the first 20 lines of .csv files of vibration data (a) from iPhone, (b) from Teensy 4.0. iPhone timestamps are absolute (based on epoch), while the teensy uses relative timestamps based on the moment each recording started (including a negligible warm up time).

Firstly, the data that was collected consists of two main types, audio data and vibration data. The audio data is in two different file formats, the data collected from the iPhone is in its native .caf format, and the audio data from the teensy is in .mp3 format. The audio from the teensy was collected in .wav format but was compressed to .mp3 format using a python script. The vibrations of the printers are both in .csv format. The data from the iPhone consists of four columns (Fig. 10). The first is the timestamp in seconds, the second is the acceleration on the x-axis, the third is the acceleration on the y-axis, and the fourth is the acceleration on the z-axis. Each column has a title, and the acceleration is measured in g's (g≈9.80665 m/s^2^). Similarly, the vibration data from the teensy has four columns with titles that are timestamped in milliseconds, x-axis acceleration, y-axis acceleration, z-axis acceleration, in that order (Fig. 10). The acceleration is measured in m/s^2^. In each experiment, the vibration and audio data collections were initiated and stopped at the same time, so the audio/vibration data pairs are synchronized within each collection platform (i.e., iPhone and teensy).

**Fig. 11:**
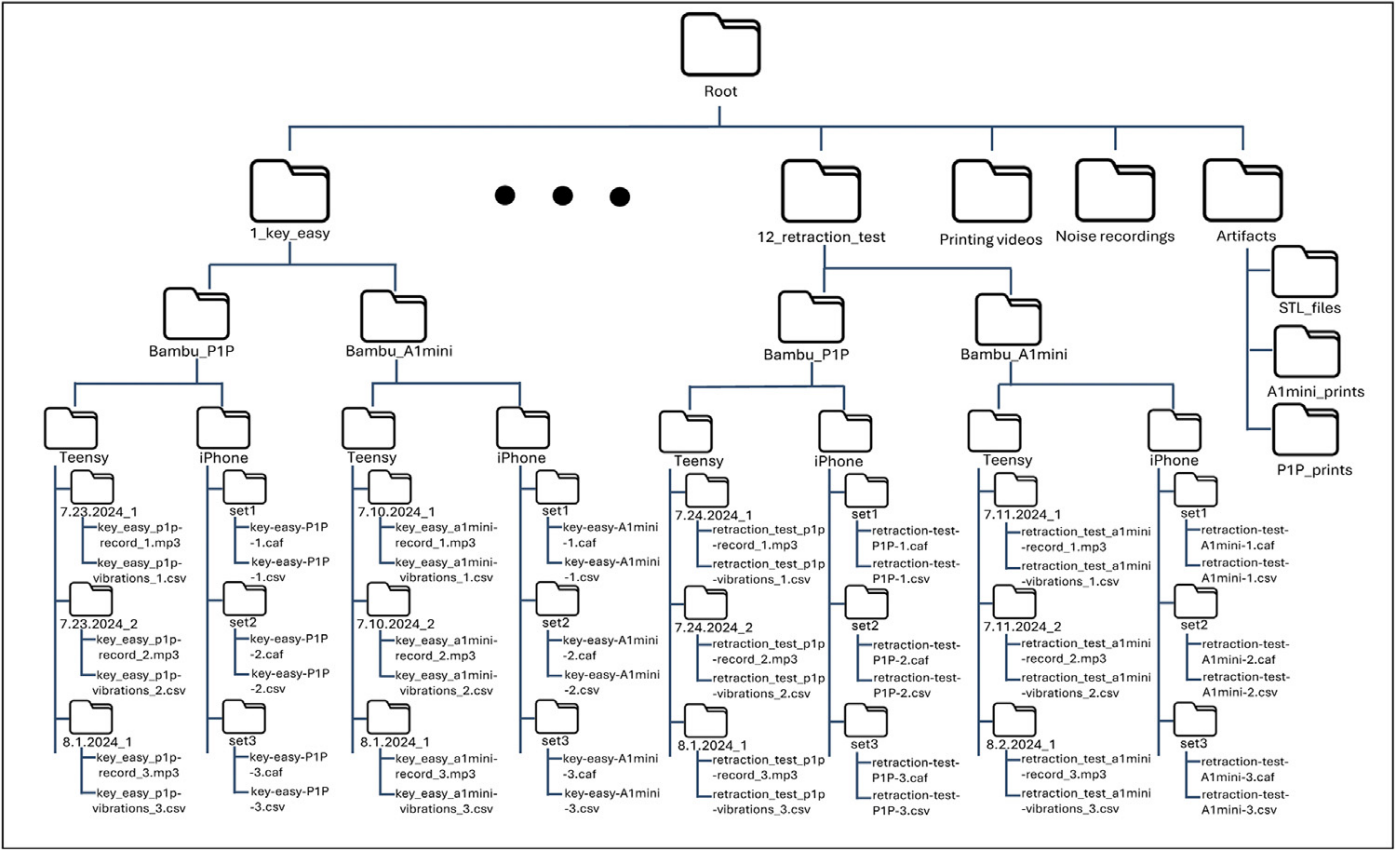
Schematic of file structure.

The entire dataset is openly accessible in the repository's data folder. Regarding the structure of the data folder, there are 144 subfolders. The root contains twelve main folders (e.g., 1_key_easy) for the artifacts and three supplementary materials such as the .3mf and .gcode files in the 3D models folder, the recordings of background noise folder, and the folder with the video recordings of prints (Fig. 11). Inside each artifact folder there are two subfolders (i.e., Bambu_P1P, Bambu_A1mini), one for each printer. Inside each printer subfolder, there are two subfolders (i.e., Teensy, iPhone), one for each data collection method. Lastly, inside each data collection method, there are three subfolders (e.g., 7.11.2024_1, set1) that contain the individual audio and vibration files. The data collected by the teensy 4.0, has folders titled with the date that the data collection happened. The iPhone data has folder names that refer to the order in which it was recorded.

### 3.4 Data characteristics

**Fig. 12:**
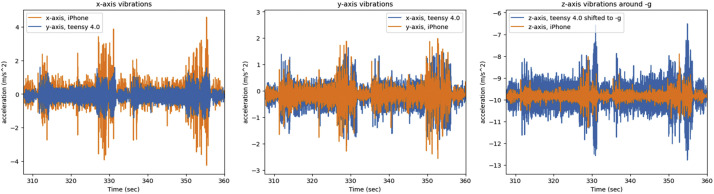
The x, y, z printer axis vibrations as recorded by both systems on the A1 mini's axes, graphed together in order to display any differences.

Furthermore, we will present some figures of the data that was collected. First, we will present a comparison of the side channel of the same artifact (*easy key*) recorded on the two different systems, the iPhone-based app, and the Teensy microcontroller system. Fig. 12 presents the comparison of the vibrations in the x, y, and z of the printer, in the figure the same two layers of the key are shown.

**Fig. 13:**
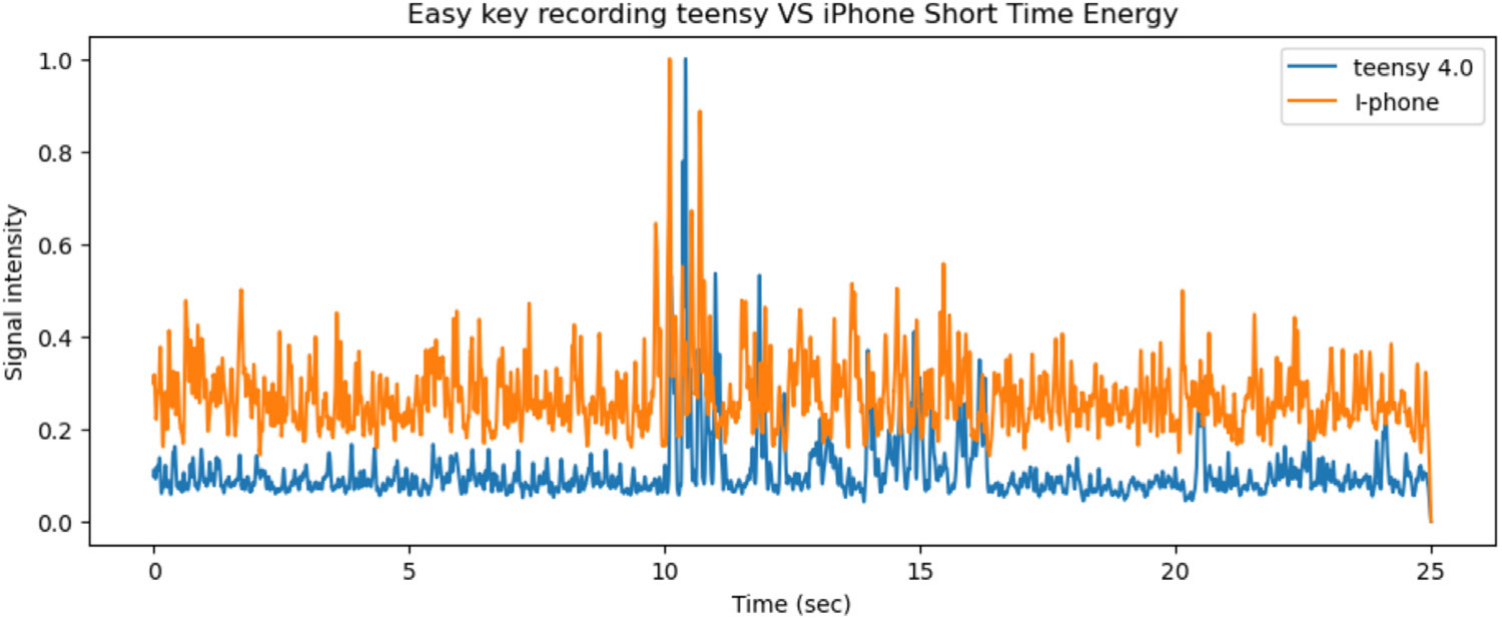
The short time energy of the iPhone and teensy microphones for the same 25 s period on the A1 mini printer.

For the audio, 25 s (335–360 s window) are taken from the A1 mini printer recordings and then the short time energy with a window length of 0.06 s is applied to both signals, the results are presented in Fig. 13. The A1 mini has active motor noise cancellation.

**Fig. 14:**
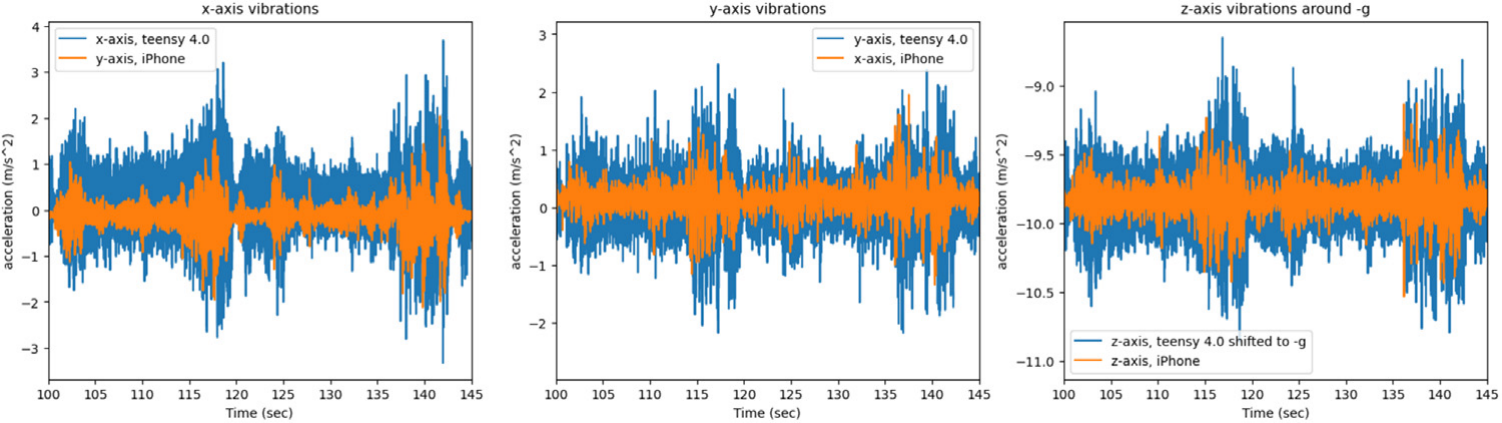
The x, y, z printer axis vibrations as recorded by both systems on the P1P's axes, graphed together to highlight any differences.

**Fig. 15:**
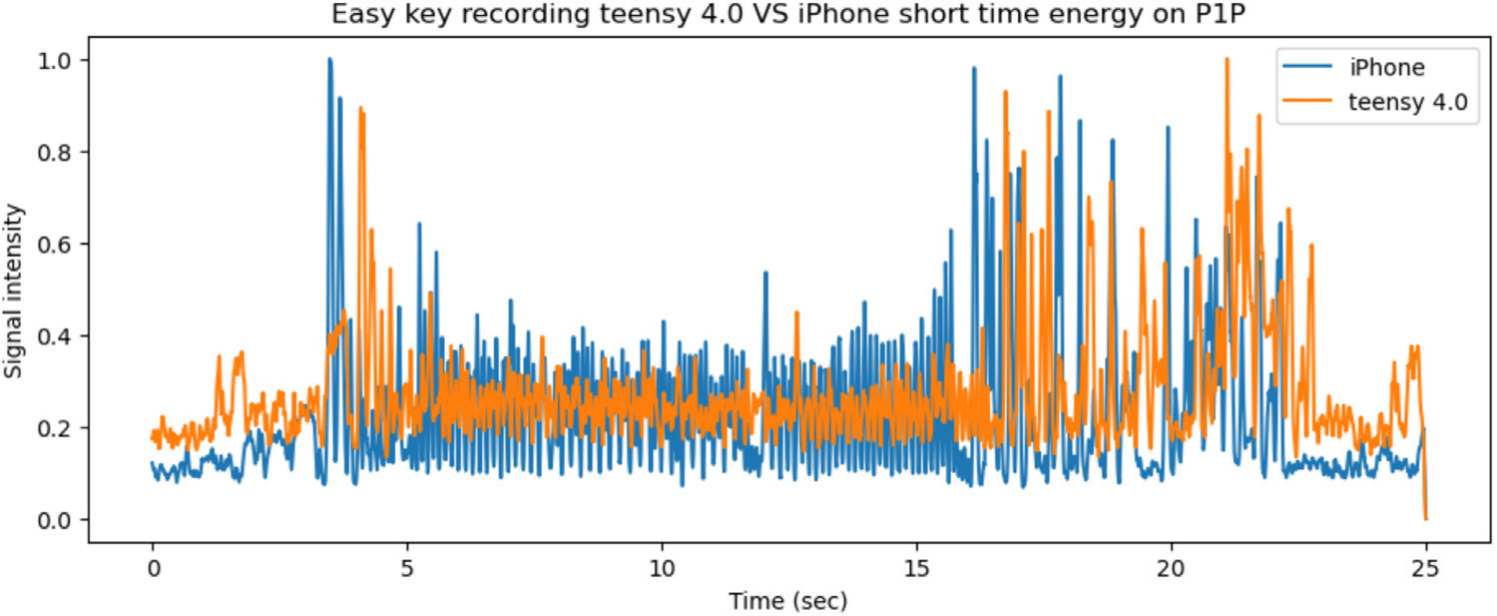
The short time energy of the iPhone and teensy microphones for the same 25 s period on the P1P printer.

Similarly, on the P1P printer the vibration and audio side channels for the same object and segments are presented in Fig. 14, Fig. 15. The 25 s period shown in Fig. 15 represents the window between 120–145 s. The P1P 3D printer has anti vibration technology.

**Fig. 16:**
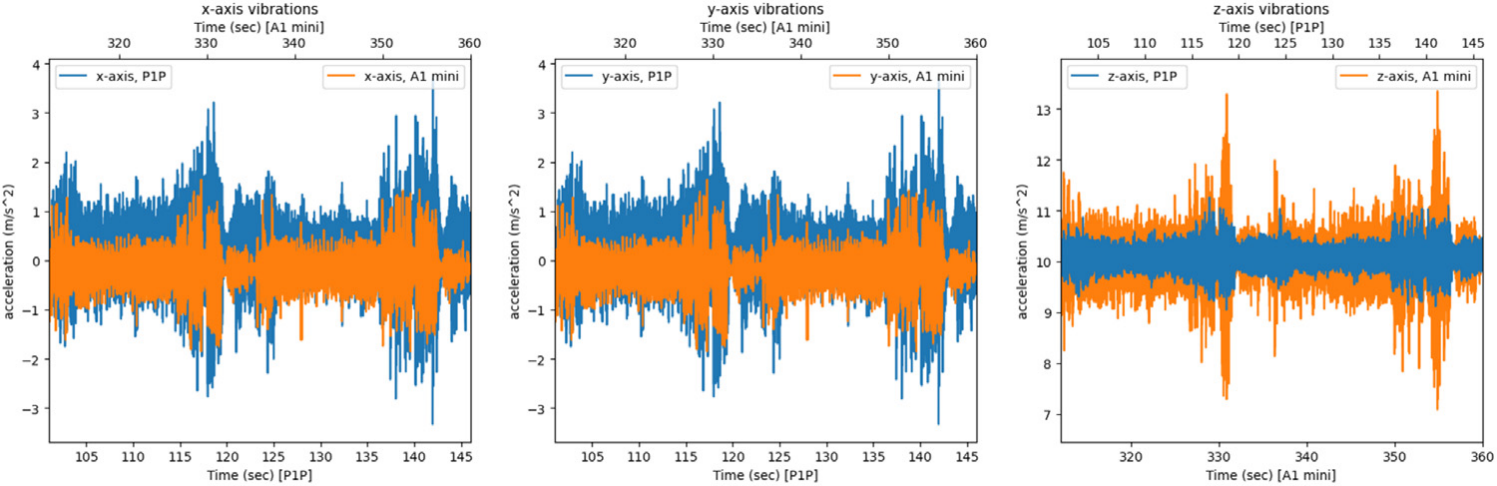
The x, y, z printer axis vibrations as recorded by the Teensy 4.0 system on both 3D printers, graphed together to highlight any differences.

**Fig. 17:**
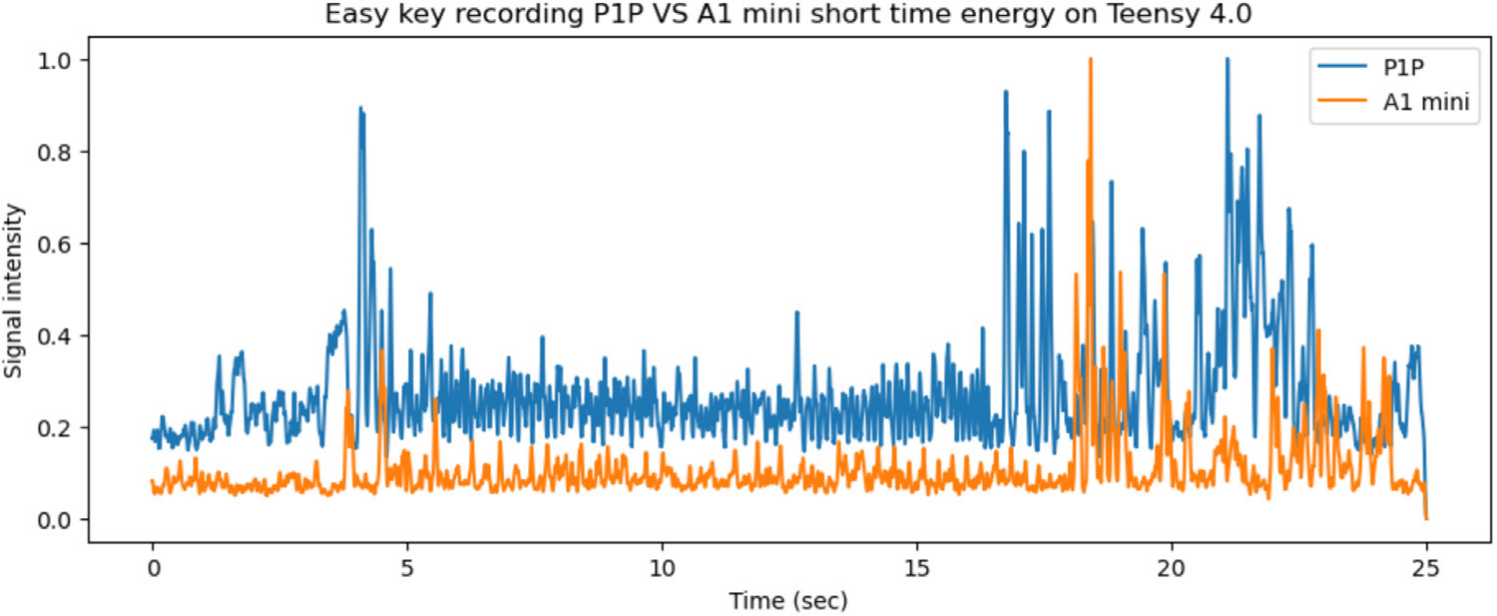
The short time energy of the A1 mini and P1P 3D printers for the same 25 s period while printing the *easy key.*

Next, the recordings for the same object on the different printers are presented. The recordings were synced in order to represent the same two layers of the *easy key* artifact. In Fig. 16, the vibrations recorded by the Teensy 4.0 system on both printers are presented. The short time energy audio signals from the two systems are compared in Fig. 17 for the same 25 s period (335–360 s window for A1 mini and 120–145 s window for P1P).

**Fig. 18:**
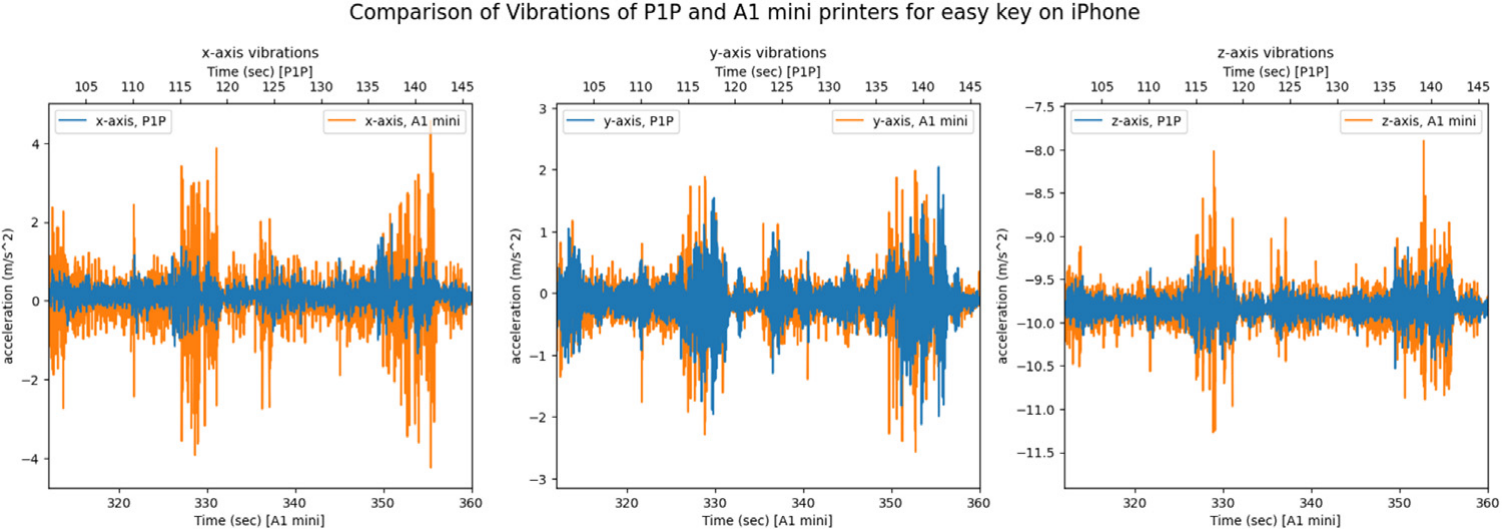
The x, y, z printer axis vibrations as recorded by the iPhone on both 3D printers, graphed together in order to highlight any differences.

**Fig. 19:**
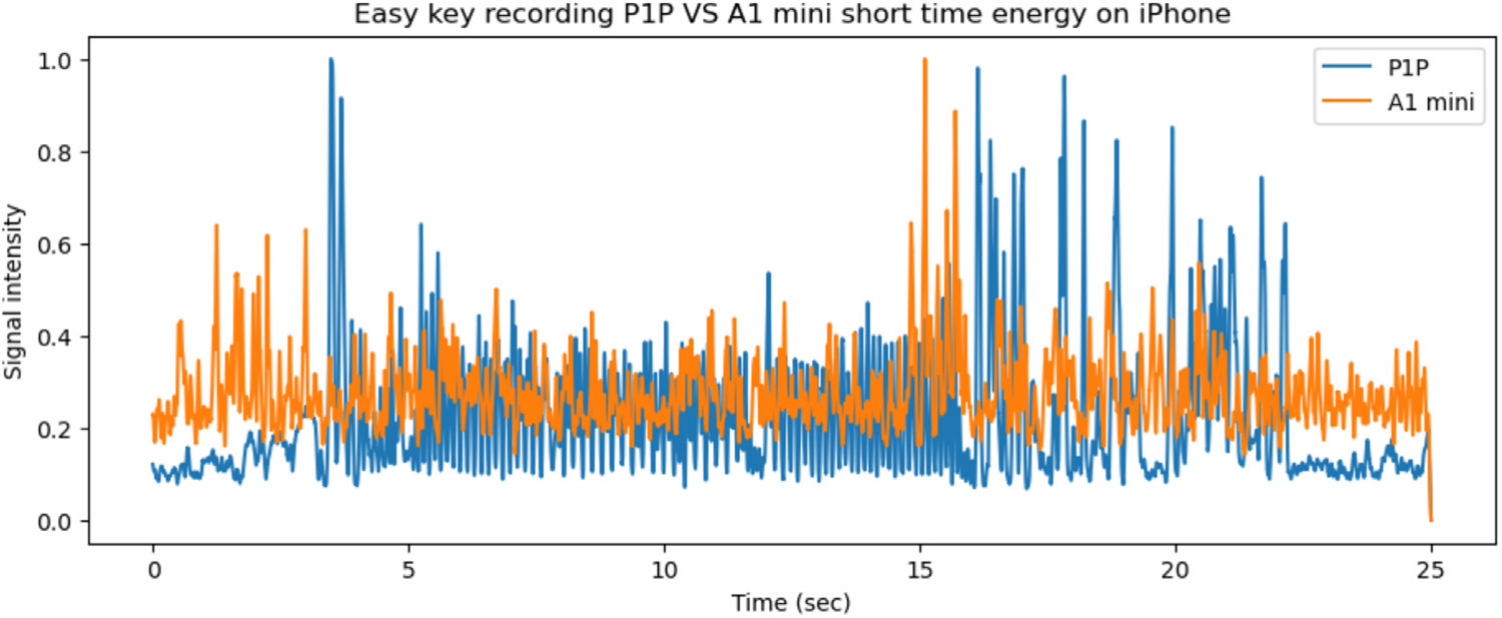
The short time energy of the A1 mini and P1P 3D printers for the same 25 s period while printing the *easy key,* recorded on the iPhone.

The same process was repeated for iPhone recordings for the *easy key* artifacts and is presented in Fig. 18, Fig. 19.

**Fig. 20:**
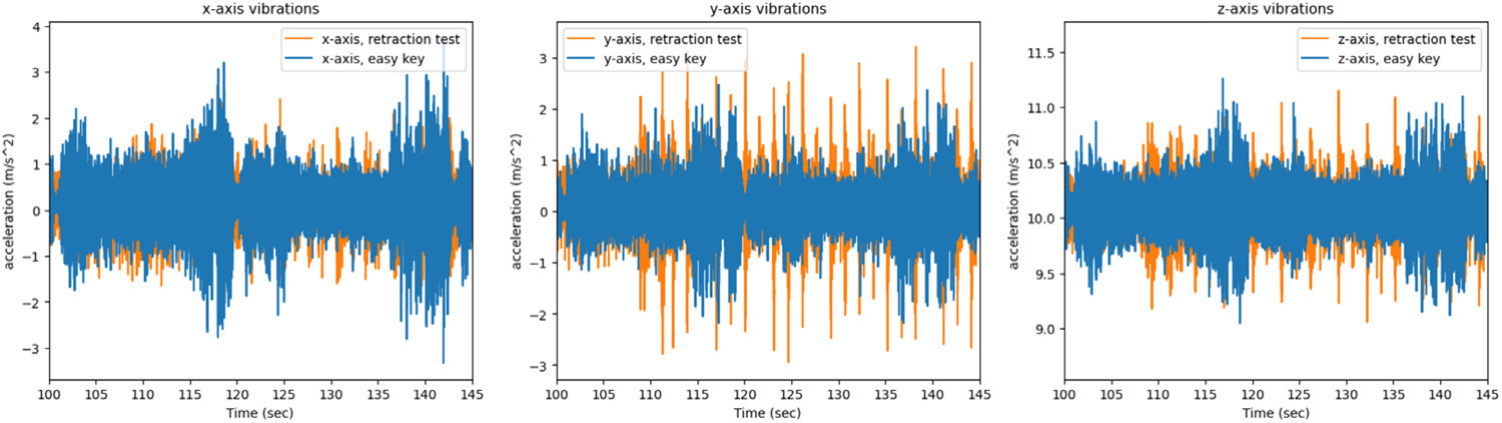
The x, y, z axis vibrations as recorded by the Teensy on the P1P for the *easy key* and *retraction test*, graphed together in order to highlight any differences.

**Fig. 21:**
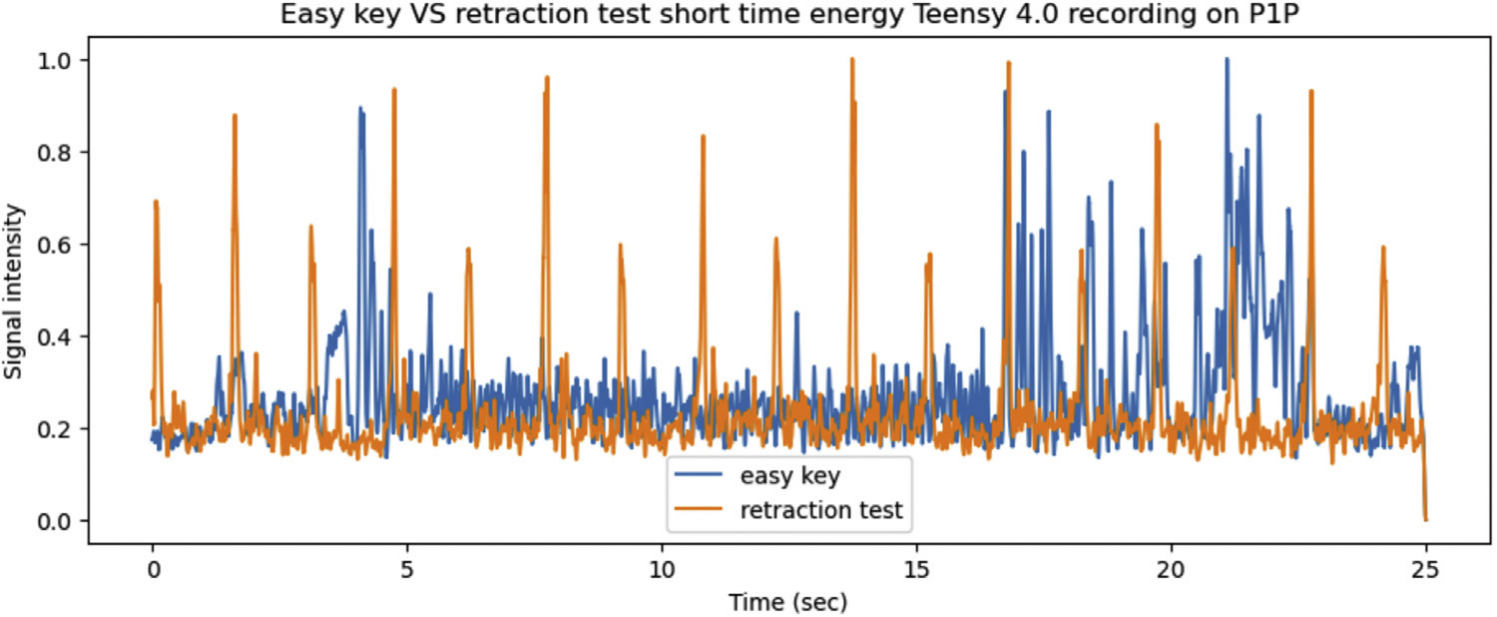
The short time energy of the *easy key* and *retraction test* for the same 25 s period while printing on the P1P.

Lasty the *retraction test* and *easy key* side channels are compared for the Teensy 4.0 on both 3D printers. First both side channels are compared for the P1P in Fig. 20, Fig. 21.

**Fig. 22:**
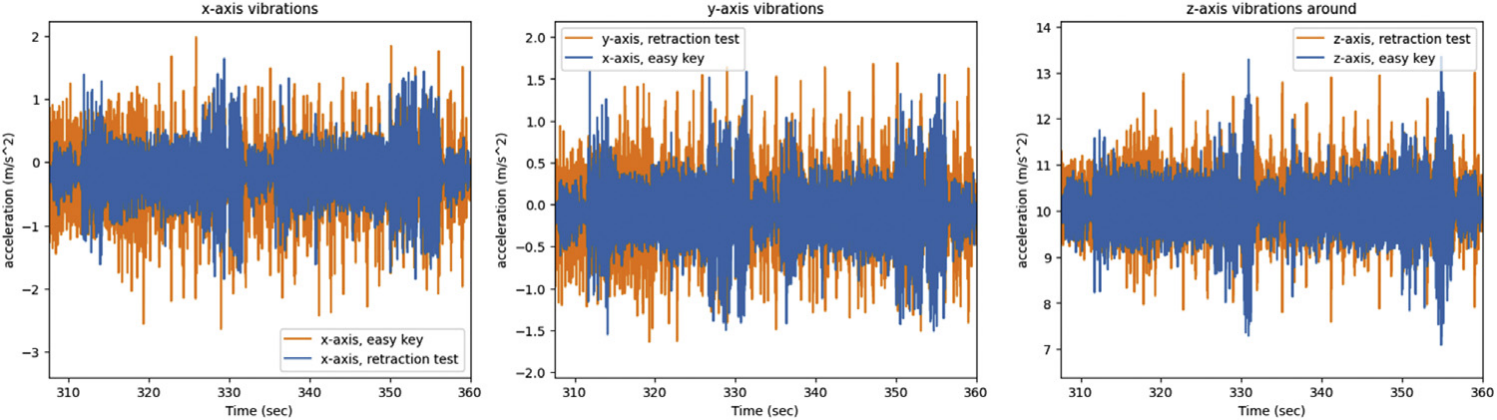
The x, y, z axis vibrations as recorded by the Teensy on the A1 mini for the *easy key* and *retraction test*, graphed together in order to highlight any differences.

**Fig. 23:**
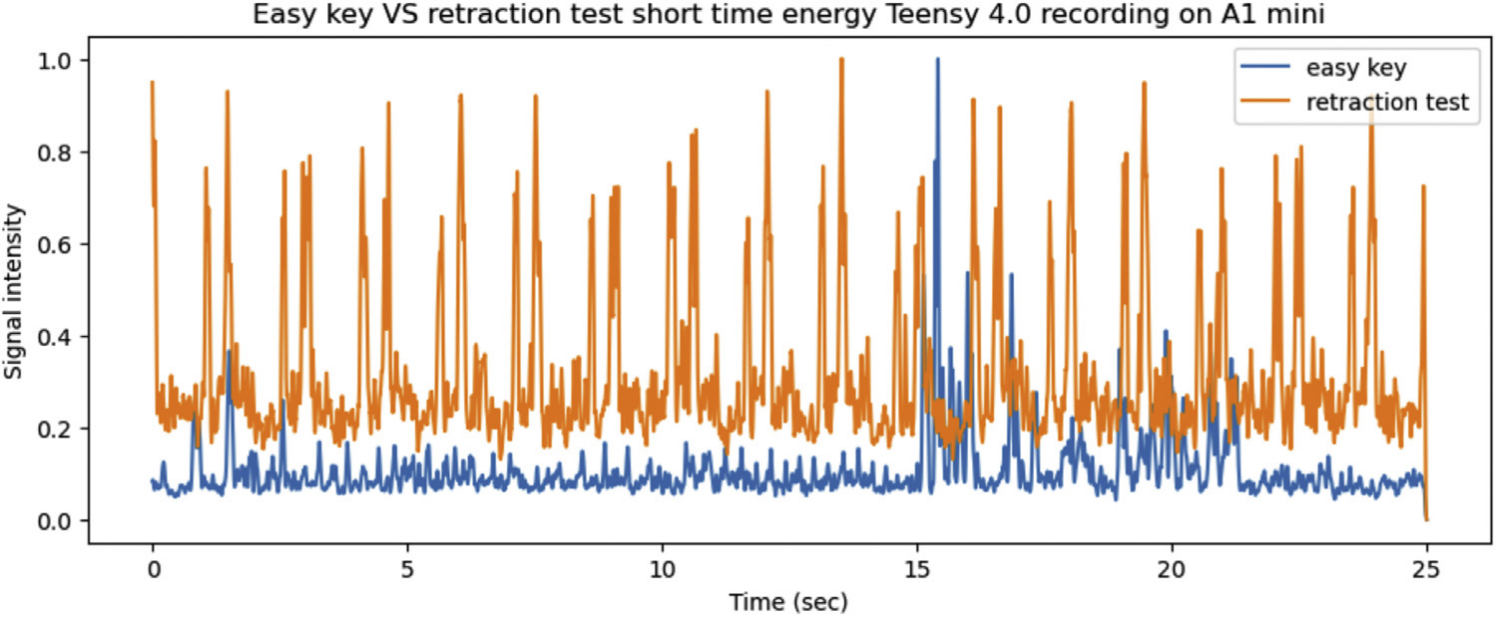
The short time energy of the *easy key* and *retraction test* for the same 25 s period while printing on the A1 mini.

The same process was repeated for the *retraction test* and *easy key* side channels are compared for the Teensy 4.0 on the A1 mini. Both side channels are compared for the A1 mini in Fig. 22, Fig. 23.

## 4 Experimental Design, Materials and Methods

The data was collected from two different 3D printers. The Bambu Labs P1P and the Bambu Labs A1 mini. The P1P is a coreXY 3D printer, with a maximum printing speed of 300 mm/s. The P1P has an automatic bed leveling sequence and vibration suppression. The vibration suppression capability is the main reason this printer was chosen. On the other hand, the A1 mini is a cartesian coordinate motion system 3D printer, with a maximum stated speed of 500 mm/s. However, for quality prints the printer uses speeds of 180–200 mm/s. The A1 mini also has an automatic bed leveling sequence and has active motor noise canceling. Both 3D printers have resonance compensation.

**Fig. 24:**
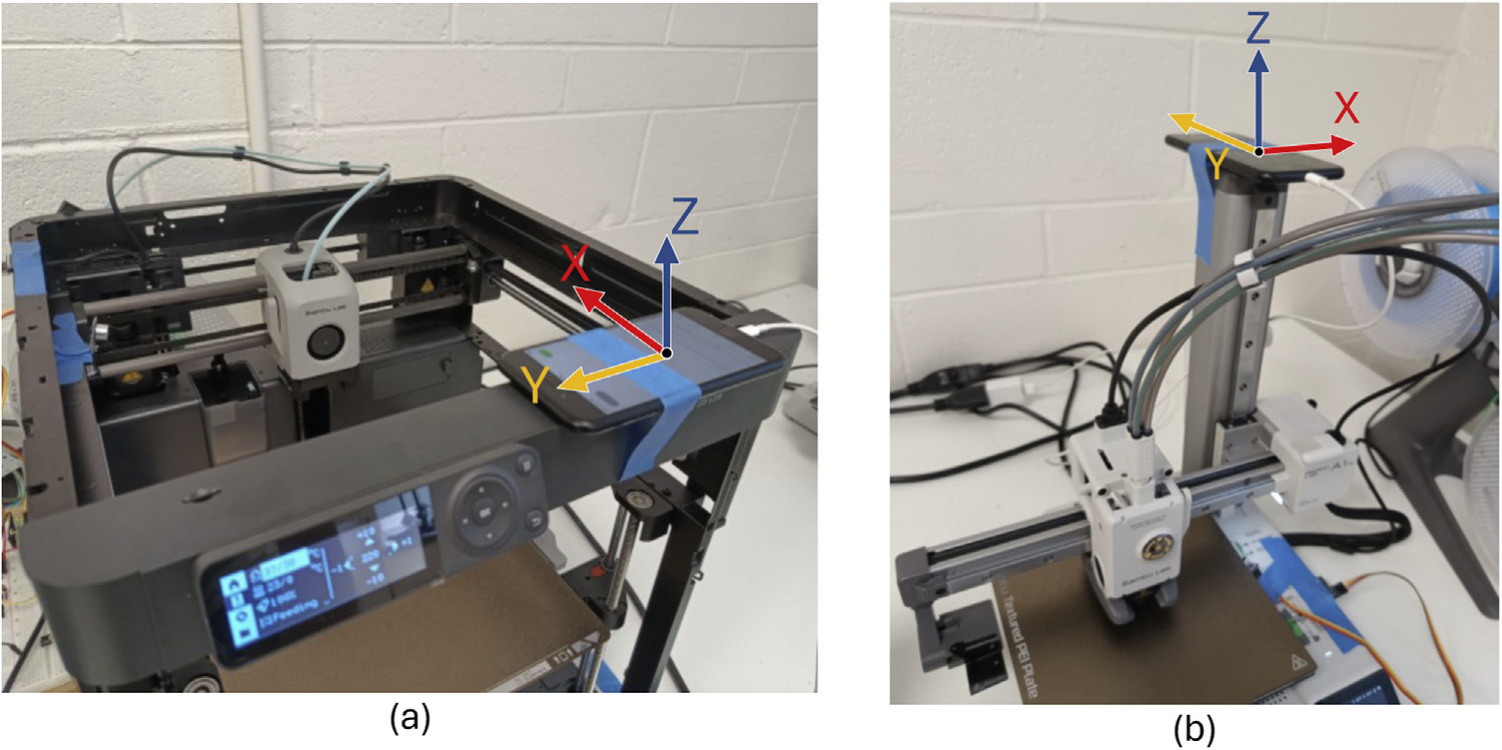
(a) The iPhone on the P1P, (b) the iPhone on the A1 mini. Both show the direction of the accelerometer x, y, and z axes.

The first method for collecting the audio and vibration data uses our CPS DataCollector App [14] on an iPhone SE (second generation). On the P1P, the iPhone is placed on the top of the printer and is secured in place using painter's tape as seen in Fig. 24. We performed tests to confirm that the painter's tape has good adhesion for placing objects on the 3D printers without slipping. On the A1 mini the iPhone is placed on the top of the z-axis pole and is secured with painter's tape. The directions of the xyz-axis system relative to the printers is shown in Fig. 24. The microphone records audio with a sampling rate of 44.1 kHz and saves it as a .caf file on the iPhone, the accelerometer records the samples at 100 Hz (± 4% error).

The second method uses a teensy 4.0 microcontroller along with an MPU 6050 gyro/accelerometer, teensy audio board, and an electret microphone on a GY-MAX4466 breakout board. The accelerometer and the audio board are directly connected to the teensy 4.0, whereas the microphone is connected to the audio board. The audio board has a slot, where a micro-SD card is placed. The recorded vibrations are saved to the SD card as a .txt file, at a rate of 500 Hz. The sound from the microphone is recorded with a sampling rate of 44.1 kHz and is also saved to the SD card in a .wav file. Once the recording is stopped, the files are loaded from the microSD card to the computer, where they are converted to .csv and .mp3 files using a python program. The teensy is powered through a USB cable connected to a laptop placed next to the printer.

**Fig. 25:**
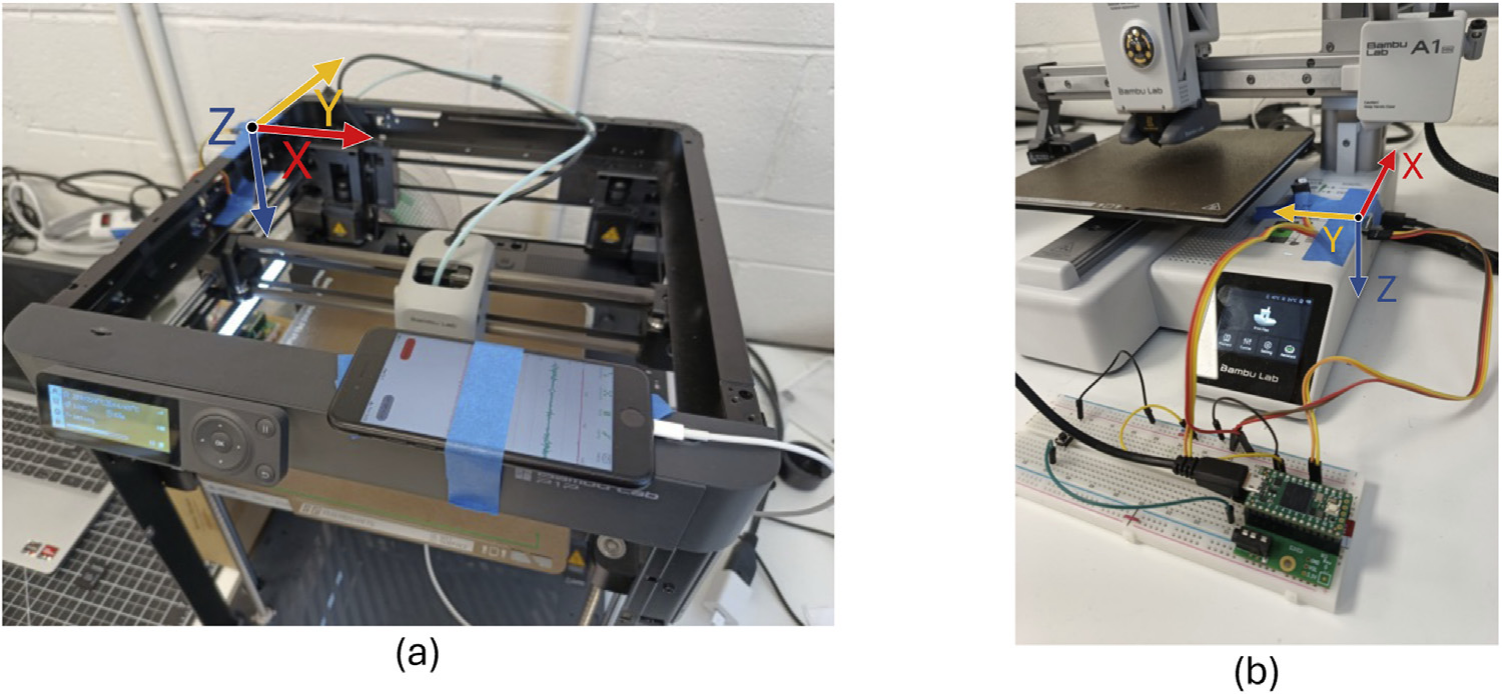
(a) The MPU 6050 on the P1P, (b) the MPU 6050 on the A1 mini. Both show the direction of the accelerometer x, y, and z axes.

The teensy and the audio board are placed on a breadboard, along with the activation button that starts and stops the recording process. The microphone sensor and the MPU 6050 are connected with wires to the breadboard, and are positioned on the printer using painter's tape. On the P1P, the sensors are placed on the top of the printer as is shown in Fig. 25. On the A1 mini, the sensors are placed on the top of the base of the printer, next to the print surface. The directions of the XYZ axes systems relative to the printers is shown in Fig. 25.

Since both printers have calibration routines, before they begin printing, the recording does not start when the print command is given to the printers. For the P1P, the recordings begin when the print head is stopped on the front left corner before it begins the printing process. The recording ends with the print head cleaning itself and the build platform being moved to 20.9 cm above the interior bottom surface of the printer enclosure. For the A1mini, the recordings begin when the printer is on the calibration of extrusion flow step of the pre-printing routine, and the recordings end with the retraction of filament step in the post printing routine. In the database as supplementary files, there are two videos of the same object (i.e. *medium key*) recording process, that show all the printer's movements before the printing of the object begins and after the printing ends that are included in all the recording data.

Our complete dataset is accessible online at [14].

## Limitations

While the data collection used two popular, commercially available 3D printers (one from each category), the data is still specific to those two machines and not to every 3D printer of similar type. Another potential limiting factor was any outside background noise, such as the air conditioning of the lab, which can be filtered using any white noise filter. The noise recordings are provided in the supplementary materials to aid in the design of a precise filter. Moreover, in our dataset, we have not applied any filtering to the collected vibration data.

## Ethics Statement

The authors have read and followed the ethical requirements for publication in Data in Brief and confirmed that the current work does not involve human subjects, animal experiments, or any data collected from social media platforms*.*

## CRediT Author Statement

**Nektarios G. Tsoutsos:** conceptualization and supervision, funding acquisition, writing - review and edit, **Christos Madamopoulos:** methodology, software, data curation, writing – review and edit.

## Data Availability

zenodo.org3D printer audio and vibration side channels (Original data).
